# Characteristics of LGV repeaters: analysis of LGV surveillance data

**DOI:** 10.1136/sextrans-2013-051386

**Published:** 2014-01-15

**Authors:** Minttu Rönn, Gwenda Hughes, Peter White, Ian Simms, Catherine Ison, Helen Ward

**Affiliations:** 1 Department of Infectious Disease Epidemiology, Imperial College London, London, UK; 2 Centre for Infectious Disease Surveillance and Control, Public Health England, London, UK; 3 Modelling and Economics Unit, Public Health England, London, UK; 4 Department of Infectious Disease Epidemiology, MRC Centre for Outbreak Analysis and Modelling, Imperial College London, London, UK; 5 Sexually Transmitted Bacteria Reference Laboratory, Public Health England, London, UK

**Keywords:** LYMPHOGRANULOMA VENEREUM, HOMOSEXUALITY, INFECTION CONTROL

## Abstract

**Objectives:**

A number of individuals have acquired lymphogranuloma venereum (LGV) infection multiple times since its re-emergence. We describe the characteristics of reinfections and those who acquire them.

**Methods:**

The LGV Enhanced Surveillance system collected detailed information on LGV episodes in the UK from 2004 to 2010. Using logistic regression we compared the baseline characteristics of men who have sex with men (MSM) who had a repeat LGV episode (‘repeaters’) to MSM with a single reported episode (‘non-repeaters’).

**Results:**

There were 66 individuals among the 1281 MSM (5.2%) with LGV episode who had a recorded reinfection during the data collection period. Those who acquired LGV reinfection were more likely to be HIV positive (97% vs 79%), visit a clinic in London (OR 2.0, 95% CI 1.1 to 3.8), and have hepatitis C (OR 2.2, 95% CI 1.1 to 4.6) or concurrent gonorrhoea (OR 2.2, 95% CI 1.2 to 3.8) on their first recorded LGV episode. Repeaters reported higher levels of unprotected sex, but behavioural variables were not significantly different between repeaters and non-repeaters.

**Conclusions:**

Among LGV repeaters, risk behaviour alone did not explain subsequent reinfection. LGV repeaters have a high level of other sexually transmitted infections (STIs) which may be linked to their central position in the sexual network that contributes to their heightened risk of STI acquisition. Given the low prevalence of LGV in the general MSM population, momentary increases in incidence in subsets of the population may be an important factor for LGV risk where the overall level of sexual risk behaviour is higher. Validating this would require research into sexual network structures.

## Introduction

Lymphogranuloma venereum (LGV) is a re-emerging STI in high-income countries. It is a biovar of *Chlamydia trachomatis* and causes a more symptomatic infection in comparison with non-LGV chlamydia.^1^ LGV is a relatively rare infection, and in the UK, it is predominantly seen in HIV-positive men who have sex with men (MSM), with an estimated 0.9% (95% CI 0.7% to 1.2%) positivity in rectum among MSM visiting genitourinary medicine (GUM) clinics.^2^

Despite its rarity, LGV has become endemic in the UK and is mainly diagnosed in HIV-positive MSM with high-risk behaviours.^3^ The LGV Enhanced Surveillance system has identified several individuals with repeat infection suggesting they have regular sexual contact with LGV-infected individuals, and potentially belong to a group which sustains LGV transmission. In classic epidemiology, those with a repeat infection have been used to explaining the persistence of STIs through infection saturation in small high-risk populations, and identifying these groups can aid a more efficient targeting of interventions.^4^ Previous studies have compared baseline characteristics of repeaters to those who do not experience a repeat infection, and this has been used to create a predictive model to estimate risk for future STIs in patients who had visited an STI clinic in Florida,^5^ and for a repeat syphilis in MSM in San Francisco.^6^ Similar analysis is presented here to explore characteristics of repeat infections using LGV Enhanced Surveillance data in the UK.

## Methods

LGV Enhanced Surveillance was a voluntary surveillance system established in response to the outbreak of LGV in the UK, and the system was managed by the STI section at the Centre for Infectious Disease Surveillance and Control in Colindale, Public Health England (formerly the Health Protection Agency). During 2004–2010, LGV Enhanced Surveillance collected demographic information about age, sex, sexuality, ethnicity, clinic location, clinical information in relation to reasons for attending, duration of symptoms, type of rectal, genital and systemic symptoms, concurrent STIs and HIV status, and behavioural information about probable country of acquisition, locations and venues used for meeting new partners, number of sexual partners, and sexual practices, and whether these were unprotected, in the past 3 months.

Episodes occurring in MSM were included in the analysis. To exclude potential treatment failures and duplicate notifications from the analysis, LGV reinfections were defined as subsequent episodes if they were recorded for the same patient at least 3 months after the first episode. Repeat patient episodes had been linked at clinic level.

To compare the baseline characteristics of repeaters and non-repeaters, we performed univariate logistic regression where possible, but if the variable's category had fewer than five events, the parameter estimate for these was not presented, and Fisher's exact test p value was calculated instead (two-sided for 2-by-2 tables, and one-sided for larger tables). All variables measured by Enhanced Surveillance were analysed in this manner, and here we present a summary of the results. A Kruskall–Wallis test was used to test differences in the median time between infections. Statistical analyses were performed in STATA SE/11.2.

## Results

Of the confirmed LGV episodes in the UK, 86.7% (1370/1581, after de-duplication) had an LGV Enhanced Surveillance form filled in. The LGV Enhanced Surveillance dataset thus had 1370 episodes of which 28 episodes occurred in females, heterosexual males, or men with unrecorded sexuality, or less than 3 months after the previous episode, and these were excluded. In this work, we looked at the remaining 1342 episodes in 1281 MSM. A total of 66 (5.2%) men were known to have been reinfected during the data collection period, and had details of at least one of their episodes in the dataset. There were 62 repeaters’ first (recorded) episodes, 59 second episodes and 6 third episodes. Of the repeaters with a first and second episode in the dataset, 46.4% went on to present with a second episode within 12 months (the overall median time to second episode was 13.2 months, with a range of 3.3–51.2 months).

We looked at the median time to reinfection by year of first infection. For those with their first episode by the end of 2005, the median time to second reinfection was 10.9 months (n=13), while for those whose first episode occurred in 2006–2007, the median was 28.4 months (n=19), for 2008–2009, 12.3 months (n=19), and for 2010, 6.3 months (n=5). Kruskall–Wallis p value was 0.003 for these categories.

We assessed potential predictors for future repeat infection by comparing the baseline characteristics (from first episode) of repeaters (n=62 with their first known episode in the dataset) to the episodes of non-repeaters (n=1215 with no known repeat infection). Due to a low number of events in the repeaters’ group, we limited the analysis to univariate level. Results are presented in table 1.

**Table 1:** Summary of variables analysed

	Non-repeaters		Repeaters’		Univariate logistic regression				Fisher's exact test
			1st episode		1st episode versus non-repeaters				p Value
	n=1215	Per cent	n=62	Per cent	OR	1.0	CI	p Value	
Age									
Mean (SD)	38.2 (8.4)	38.8 (9.2)	1.0	1.0	1.0	0.618			
HIV status									
Negative/unknown	252	20.7	2	3.2	N/A				<0.001
positive	963	79.3	60	96.8					
Seen in a clinic in London									
No	394	32.4	12	19.4	1.0				
Yes	821	67.6	50	80.7	2.0	1.1	3.8	0.034	
Presentation year									
2010	847	70.2	54	87.1	1.0				
<2010	360	29.8	8	12.9	0.3	0.2	0.7	0.006	
No other STIs									
No	456	37.5	31	50.0	1.0				
Yes	691	56.9	27	43.6	0.6	0.3	1.0	0.040	
Unknown	68	5.6	4	6.5	N/A				
Gonorrhoea									
No	950	78.2	40	64.5	1.0				
Yes	198	16.3	18	29.0	2.2	1.2	3.8	0.009	
Unknown	67	5.5	4	6.5	N/A				
Hepatitis C (PCR)									
No	422	34.7	21	33.9	1.0				
Yes	110	9.1	12	19.4	2.2	1.0	4.6	0.038	
Unknown	683	56.2	29	46.8	0.9	0.5	1.5	0.588	
Hepatitis C (Ab)									
No	800	65.8	35	56.5	1.0				
Yes	168	13.8	17	27.4	2.3	1.3	4.2	0.006	
Unknown	247	20.3	10	16.1	0.9	0.5	1.9	0.832	
RAI									
None reported	75	6.2	0	0.0	N/A				
Reported protected/=protection unknown	225	18.5	9	14.5	1.0				
Unprotected	810	66.7	51	82.3	1.6	0.8	3.2	0.219	
Unknown	105	8.6	2	3.2	N/A				
IAI									
None reported	109	9.0	4	6.5	N/A				
Reported protected/protection unknown	204	16.8	9	14.5	1.0				
Unprotected	642	52.8	40	64.5	1.4	0.7	3.0	0.361	
Unknown	260	21.4	9	14.5	0.8	0.3	2.0	0.614	
Any oral sex									
None reported	98	8.1	1	1.6	N/A				0.199*
Reported some†	64	5.3	2	3.2					
Both receptive and insertive, unprotected	869	71.5	51	82.3					
Some or all unknown	184	15.1	8	12.9					
Any fisting									
None reported	485	39.9	26	41.9	N/A				0.639*
Reported some†	54	4.4	3	4.8					
Both receptive and insertive, unprotected	64	5.3	5	8.1					
Some or all unknown	612	50.4	28	45.2					
Sharing sex toys									
No	478	39.3	23	37.1	1.0				
Any	79	6.5	9	14.5	2.4	1.1	5.3	0.036	
Unknown	658	54.2	30	48.4	1.0	0.5	1.7	0.849	

Comparisons are between repeaters' first episode and non-repeaters.

N/A, not applicable (parameter estimate not presented due to low number of events in the cell).

*One-sided p value for Fisher's exact test (for tables larger than 2-by-2), cut-off value for statistical significance in this case 0.025.

†Reported some: reported either insertive or receptive (protected, unprotected or protection unknown), but did not report both unprotected.

IAI, insertive anal intercourse; RAI, receptive anal intercourse.

Repeaters’ baseline episode was associated with being HIV positive at the first LGV episode (Fisher's exact p value<0.001). Repeaters’ were twice as likely to be diagnosed in London (OR 2.0, 95% CI 1.1 to 3.8) and have a concurrent gonorrhoea diagnosis as non-repeaters (OR 2.2, 95% CI 1.2 to 3.8), and less likely to have no other STI coinfection (OR 0.6 95% CI 0.3 to 1.0). Current hepatitis C infection (PCR positive) and hepatitis C antibody positivity were over twice as likely to occur among repeaters. The proportion of men who were hepatitis C PCR positive increased from 19.4% (12/62) on a repeater's first episode to 27.1% (16/59) and 50.0% (3/6) on a repeater's second and third episodes, respectively.

There was an overall trend of repeaters reporting more unprotected sex than non-repeaters, including receptive and insertive anal intercourse, unprotected oral sex, unprotected fisting and sharing of sex toys. However, the difference was not statistically significant for behavioural variables except sharing of sex toys.

## Discussion

We have described characteristics for LGV reinfection in the LGV Enhanced Surveillance data in the UK. The median time to second infection varies by the year of first infection. This probably reflects the retrospective nature of the dataset with later episodes having a shorter follow-up time to acquire LGV again. An alternative reason for changing patterns of reinfection could be increased risk during periods of increasing incidence, but this is difficult to estimate given the dataset does not actively follow-up on people's infection status.

Comparing baseline data of repeaters with non-repeaters showed that repeaters reported more unprotected sex, although the overall level of unprotected sex was high in both groups. At baseline, repeaters were significantly more likely to be HIV positive and be diagnosed in London, and have concurrent gonorrhoea and hepatitis C infection compared with non-repeaters. These may be proximate determinants for the LGV prevalence in the partner pool. Past STI infection is a predictor of future STIs. In STI clinic-based study in San Diego,^7^ history or current diagnosis of gonorrhoea or chlamydia were predictive of subsequent STI diagnosis. The more STIs the patient reported, the higher the risk for subsequent STI, which was interpreted as indicating that these people were central to sexual networks where gonorrhoea and chlamydia are transmitted. Previous contact tracing has indicated an overlap between sexual networks which transmit LGV and hepatitis C.^8^

This study is limited by a small sample size of reinfections which constrained the analysis to a univariate level, and prohibits the use of robust predictive models as well as reducing the statistical power of the study. If heightened risk behaviour had an association with repeated LGV acquisition, such as repeaters having continued risk behaviour while non-repeaters had transient risk behaviour prior to LGV acquisition, we would not be able to observe this in the cross-sectional dataset. In a Dutch study based in an STI clinic in Amsterdam,^9^ they found 12.6% (46/365) of MSM with an anorectal LGV infection to subsequently acquire a reinfection, which suggests that our estimate of 5.2% (66/1281) of MSM acquiring LGV more than once to be an underestimate. The repeaters were identified at clinic level, and we did not have information on how many patients were ‘lost to follow-up’ (by visiting a different clinic). Misclassification of repeaters as non-repeaters was likely if they visited a different clinic. This would dilute the association seen, unless the exposure variable and probability of being detected as a repeater are associated. HIV-positive individuals have increased contact with the healthcare system, and they may be more likely to be identified as a repeater due to this.

## Conclusions

This investigation further supports the idea of LGV being confined to dense sexual networks of mainly HIV-positive MSM. Risk for reinfection is also determined by extrinsic factors, mainly by the prevalence of infection. As LGV is still a rare infection, stochastic events are likely to play a role. Those who acquired LGV reinfection were more likely to have concurrent gonorrhoea, and more importantly, hepatitis C. Concurrent STIs are of clinical importance, and are the clearest indicator of a patient's elevated risk for future LGV infections and other STIs. Increased frequency of testing may be the appropriate means of infection management in these cases without forgetting the need of sexual health counselling.

**Key messages**

- Those who have been reinfected with LGV were more likely to have acquired other STIs, most notably gonorrhoea and hepatitis C.
- LGV repeaters did not report significantly more unprotected sex than LGV non-repeaters. Other factors, such as sexual network position, are likely influential for reinfection risk.
- In settings where LGV prevalence is higher, such as in London and among HIV-positive MSM, risk for reinfection was elevated.
